# Carbon Black-Carbon Nanotube Co-Doped Polyimide Sensors for Simultaneous Determination of Ascorbic Acid, Uric Acid, and Dopamine

**DOI:** 10.3390/ma11091691

**Published:** 2018-09-12

**Authors:** Yue Wang, Tian Yang, Yasushi Hasebe, Zhiqiang Zhang, Dongping Tao

**Affiliations:** 1 School of Chemical Engineering, University of Science and Technology Liaoning, 185 Qianshan Middle Road, High-Tech Zone, Anshan 114051, Liaoning, China; yt851991425@163.com (T.Y.); zhangzhiqiang@ustl.edu.cn (Z.Z.); 2 Department of Life Science and Green Chemistry, Faculty of Engineering, Saitama Institute of Technology, 1690 Fusaiji, Fukaya, Saitama 369-0293, Japan; hasebe@sit.ac.jp; 3 School of Mineral Engineering, University of Science and Technology Liaoning, 185 Qianshan Middle Road, High-Tech Zone, Anshan 114051, Liaoning, China

**Keywords:** polyimide, simultaneous determination, uric acid, dopamine, ascorbic acid

## Abstract

Carbon black (CB) and carbon nanotube (CNT) co-doped polyimide (PI) modified glassy carbon electrode (CB-CNT/PI/GCE) was first prepared for the simultaneous determination of ascorbic acid (AA), dopamine (DA), and uric acid (UA). The CB-CNT/PI/GCE exhibited persistent electrochemical behavior and excellent catalytic activities. Cyclic voltammetry (CV) and differential pulse voltammetry (DPV) were used for the simultaneous detection of AA, DA, and UA in their ternary mixture. The peak separations between AA and DA, and DA and UA, are up to 166 mV and 148 mV, respectively. The CB-CNT/PI/GCE exhibited high sensitivity to DA and UA, with the detection limit of 1.9 µM and 3 µM, respectively. In addition, the CB-CNT/PI/GCE showed sufficient selectivity and long-term stability, and was applicable to detect AA, DA, and UA in human urine sample.

## 1. Introduction

Dopamine (DA) is an important catecholamine neurotransmitter for message transfer in the mammalian central nervous system [1]. It plays an important role to regulate physiological processes and has direct effects on human emotion [2,3,4]. Low concentration level of DA is associated with neurological disorders such as Parkinson’s disease, schizophrenia, and Huntington’s disease [5]. Uric acid (UA) is the primary end product of purine metabolism that can be found in different concentrations in urine and human blood plasma [6]. The abnormal concentration level of UA will lead to several diseases such as leukemia, pneumonia [7], hyperuricemia, and gout [8]. Ascorbic acid (AA) exists in the human body, prevents scurvy, and is known to participate in several biological reactions. The accurate and rapid determination of these three species is highly demanded in the medical diagnosis. However, UA, AA, and DA always coexist in real biological samples. In addition, they show nearly the same oxidation potential on traditional electrodes [9]. Therefore, the development of a selective method for the simultaneous detection of the mixture is highly desirable [10].

On account of the comprehensive consideration of in vivo environment, it is necessary to develop a simultaneous, rapid, and accurate analytical method for the determination of the ternary mixture of AA, DA, and UA. Electrochemical techniques are the most used method for the quantitative determination of AA, DA, and UA with excellent accuracy. Numerous materials such as carbon nanotube [11,12], graphene [13,14,15], graphene oxide [16], MoS_2_ [2,9] metal and metal oxide [17,18,19], conducting polymer [20,21], boron-doped diamond [22], silica gel [23], and nafion [24] have been used for the modification of electrodes to realize the simultaneous detection of AA, DA, and UA. Polyimide (PI) is considered an ideal polymer material due to its high thermal stability, excellent chemical and physical properties, and good adhesion properties [25]. Typically, PI is widely used in the semiconductor industry for passivation or dielectric layer [26]. The highly-insulating property of PI, however, restricts the application in electronic devices of PI.

In this study, we suggest a method to fabricate a thin PI composite film on glassy carbon electrode (GCE). The PI was doped with the hybrid material of carbon black (CB) and carbon nanotube (CNT). By taking advantages of high electronic conductivity of CB and CNT, the CB-CNT/PI composite exhibits excellent responses to the simultaneous detection of AA, DA, and UA. Owing to the desirable structure and stability of PI, the present CB-CNT/PI/GCE exhibits good selectivity and long-term stability. 

## 2. Materials and Methods

### 2.1. Reagents and Materials

The reagent 4,4′-(Hexafluoroisopropylidene) diphthalic dianhydride (6FDA) was purchased from ChinaTech (Tianjin, China) Chemical Co. Ltd., which was recrystallized from acetic anhydride, and vacuum dried before using. The reagent 9,9′-bis(4-hydroxy phenyl) fluorene (BHF) was purchased from Sinosteel Anshan Research Institute of Thermo-energy Co. Ltd., Anshan, China. *N*,*N*′-dimethylacetamide (DMAc), Pd/C (10%), hydrazine hydrate, acetone, nitric acid, ethanol, acetic anhydride, pyridine, K_2_HPO_4_, KH_2_PO_4_, uric acid (UA), ascorbic acid (AA) and dopamine (DA), urea, glucose, fructose, maltose, hydrogen peroxide (H_2_O_2_), potassium chloride (KCl), ironic chloride (FeCl_3_), ammonium chloride (NH_4_Cl), sodium sulphate (Na_2_SO_4_), sodium chloride (NaCl), and citric acid were purchased from Sinopharm Chemical Reagent Co., Ltd., Beijing, China. DMAc was dried using anhydrous molecular sieves before using. Carbon black (CB) was obtained from CABOT Corporation and used as received. Carbon nanotube (CNT) was obtained from Showa Denko Co. Ltd., Tokyo, Japan. Glassy carbon electrode (GCE, 3.0 mm in diameter) was obtained from Shanghai Chen Hua Instrument Co. Ltd., Shanghai, China. A 0.1 M phosphate buffer solution (PBS, prepared by using K_2_HPO_4_ and KH_2_PO_4_) was used to prepare electrolyte. Doubly distilled water was used throughout the experiments. All other chemicals were of analytical grade and were used without further purification.

### 2.2. Apparatus

The X-ray diffraction (XRD) pattern was taken from 5° to 60° (2θ value) with Cu-Kα radiation (λ = 1.541 Å) by an X’ Pert Powder X-ray diffractometer (X’Pert PRO, PANalytical, Almelo, The Netherlands). Fourier transform infrared spectrometry (FT-IR) spectra were measured using a Nicolet IS 10 (Thermo Scientific, Madison, WI, USA) equipped in the range of 500–4000 cm^−1^, the average scanning frequency of the instrument was 16 times. The synthesized monomer and polyimide were analyzed by proton nuclear magnetic resonance (^1^H-NMR) spectra on a Bruker Advance-AV 500 MHz instrument (Bruker, Karlsruhe, Germany) in deuterated dimethyl sulfoxide. The morphologies of the modified electrode surfaces were observed using the field emission scanning electron microscope (FE-SEM, ΣIGMA-HD, ZEISS, Oberkochen, Germany). Electrochemical measurements were performed with a CHI 750D workstation (Shanghai Chenhua, Shanghai, China). A conventional three electrode system with PI modified GCE as working electrode, a 1.0 mm diameter of Pt wire as counter electrode, and Ag/AgCl (sat. KCl) as reference electrode was employed in this study. All measurements were performed in air at room temperature of approximately 20 °C.

### 2.3. Synthesis of PI

#### 2.3.1. The Synthesis of 9,9′-Bis[4-(4-nitro-2-hydroxybenzoxyl) phenyl] Fluorene (BNHOPF)

The BHF (1 mol) was reacted with 5-fluoro-2-nitrophenol (2 mol) by nucleophilic aromatic substitution in the presence of potassium carbonate (2 mol) and DMAc under 12 h of heating. The mixture was poured into distilled water for cooling, the yellow solid compound was filtered and dried. (HPLC: 99.8%, yield: 80.25%, melting point: 185.2 °C). The detailed reaction procedure is shown in the first step of Figure S1. Figure S2 is the ^1^H-NMR spectrum of BNHOPF (δ 11.14 (s, 2H), 7.97 (td, *J* = 6.1, 3.0 Hz, 4H), 7.52 (d, *J* = 7.7 Hz, 2H), 7.45 (t, *J* = 7.4 Hz, 2H), 7.38 (s, 2H), 7.23 (dd, *J* = 8.7, 2.0 Hz, 4H), 7.14–7.07 (m, 4H), 6.52 (dd, *J* = 9.1, 7.2 Hz, 4H).

#### 2.3.2. The Synthesis of 9,9′-Bis[4-(4-amino-2-hydroxybenzoxyl) phenyl] Fluorene (BAHOPF)

Thirty milliliters of ethanol, 0.01 mol (BNHOPF), and 0.25 g Pd/C were mixed in a three-necked flask. Twenty milliliters of hydrazine hydrate were dropped into the three-necked flask with a constant pressure funnel within 1 h. The reaction was fulfilled after heating for 12 h. The white crystals were obtained after recrystallization (HPLC: 99.2%, yield: 87.5%, melting point: 280.3 °C). The reaction procedure is shown in the second step of Figure S1. Figure S3 is the ^1^H-NMR spectrum of BAHOPF (δ 9.25 (s, 2H), 7.91 (d, *J* = 7.5 Hz, 2H), 7.49–7.36 (m, 5H), 7.31 (t, *J* = 7.5 Hz, 3H), 7.03 (d, *J* = 8.5 Hz, 5H), 6.75 (d, *J* = 8.4 Hz, 5H), 6.55 (d, *J* = 8.3 Hz, 2H), 6.34 (d, *J* = 2.5 Hz, 2H), 6.26 (dd, *J* = 8.5, 2.5 Hz, 2H)).

#### 2.3.3. Preparation of Polyimide (PI)

The preparation process of polyimide is shown in Figure S4. Briefly, BAHOPF (10 mmol) was dissolved in DMAc in a three-necked flask under nitrogen atmosphere. Subsequently, the reaction mixture was cooled to 0 °C until the solid dissolved entirely. Then, 6FDA (10 mmol) was added immediately to the mixture and stirred for 12 h in N_2_ atmosphere to obtain a viscous poly (amic acid) solution. Finally, the solution was poured into methanol to obtain a white precipitate, which was collected by filtration and vacuum dried at 150 °C for 12 h.

#### 2.3.4. Fabrication Procedures of the CB-CNT/PI/GCE

**Scheme 1:**
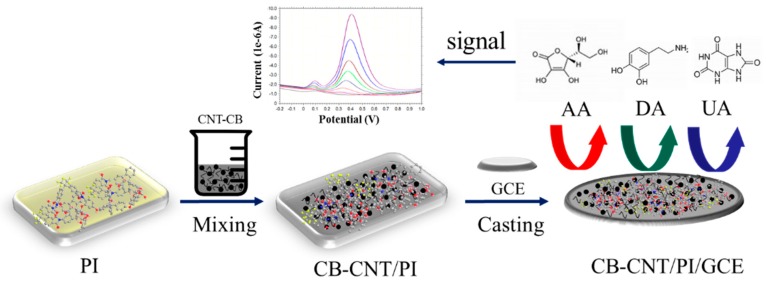
Schematic representation of the fabrication of carbon black-carbon nanotube (CB-CNT)/polyimide (PI) modified glassy carbon electrode (GCE) for the detection of ascorbic acid (AA), dopamine (DA), and uric acid (UA).

PI (30 mg) was diluted in DMAc solvent (0.57 g) and sonicated for 30 min to make a homogeneous solution. The CB solution was prepared by putting 15 mg of CB into 0.5 mL DMAc and sonicated for 30 min. The CNT solution was prepared by putting 15 mg of CNT into 0.5 mL DMAc and sonicated for 30 min. Then the CB solution was mixed with CNT solution (v:v = 1:1) and sonicated for 30 min. The PI solution was mixed with the CB-CNT mixture and sonicated for 30 min which donated as CB-CNT/PI solution. The GCE surface was polished successively with 1.0, 0.3, 0.05 µm α-alumina slurries to make a shiny surface. The cleaned GCE was rinsed and sonicated with distilled water and ethanol to remove any adhering alumina. Then the CB-CNT/PI solution (10 µL) was dropped onto the GCE surface for 12–24 h to prepare the sensor. The fabrication of CB-CNT/PI/GCE and the detection of the substances with the electrochemical detection process is shown in Scheme 1.

## 3. Results and Discussion

### 3.1. Structure Characterization of Monomers

The structures of BNHOPF and BAHOPF were confirmed by FT-IR. Figure S5 is the FT-IR spectra of dinitro compound (BNHOPF, Figure S5a) and diamine compound (BAHOPF, Figure S5b). The disappearance on typical absorption peaks of dinitro group (1585 cm^−1^ and 1330 cm^−1^) and the appearance of characteristic absorption peaks of amine groups (3382 cm^−1^ and 3318 cm^−1^) are good evidence for the successful reduction reaction.

### 3.2. Structure Characterization of Polyimide Powder

FT-IR spectra of PI was investigated (Figure S6). The peaks around 1792 cm^−1^, 1714 cm^−1^, 1383 cm^−1^, and 722 cm^−1^ correspond to asymmetric and symmetric imide ring stretching vibrations of C=O, C–N vibration, and C=O bending vibration, respectively. The broad peak around 3400 cm^−1^ corresponds to the asymmetric stretching of O–H bond. [27] Furthermore, in the wide-angle X-ray scattering patterns, the polyimides exhibit one main diffraction peak at d = 0.524 nm (2θ = 16.89 °) as shown in Figure S7. The d-spacing is usually considered to represent the distance between segments of different chains.

### 3.3. Characterization of the CB-CNT/PI/GCE

**Figure 1:**
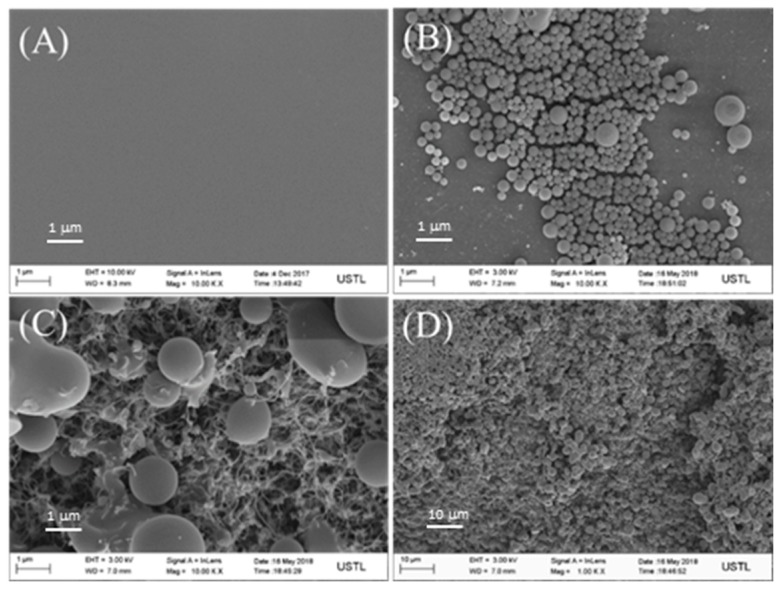
SEM images of polyimide (PI)/ glassy carbon electrode (GCE) (**A**), carbon black (CB)/PI/GCE (**B**), and CB-carbon nanotube (CNT)/PI/GCE (**C**,**D**).

The SEM technique was applied to study the morphology of the CB-CNT/PI/GCE (Figure 1). The PI-modified GCE surface shows relatively flat and smooth morphology (Figure 1A). As compared with CB/PI modified GCE (Figure 1B), co-doped with CNT enhances the dopant amount, as well as the homogeneity and dispersion of dopants (Figure 1C,D). Hence, the improvement of the electrochemical properties can be expected by the doping of CB and CNT.

### 3.4. Electrocatalytic Oxidation of AA, DA, and UA

**Figure 2:**
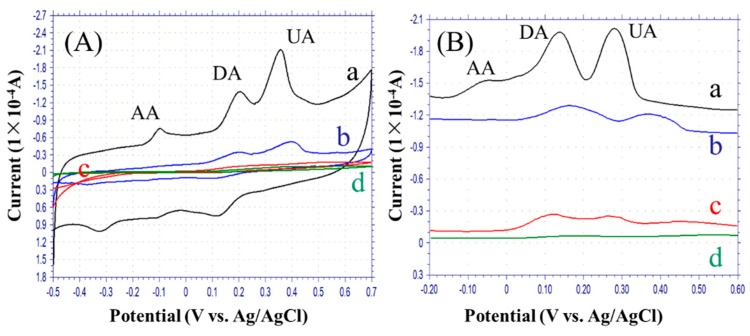
(**A**) Cyclic voltammetry (CV) responses of ternary mixture of 1.0 mM ascorbic acid (AA), 1.0 mM dopamine (DA), and 1.0 mM uric acid (UA) in 0.1 M phosphate buffer solution (PBS) (pH 7.0) on CB-CNT/PI/GCE (a), CB/PI/GCE (b), CNT/PI/GCE (c), and PI/GCE (d) at 5 mV/s; (**B**) differential pulse voltammetry (DPV) responses of ternary mixture of 1.0 mM AA, 1.0 mM DA, and 1.0 mM UA in 0.1 M PBS (pH 7.0) on CB-CNT/PI/GCE (a), CB/PI/GCE (b), CNT/PI/GCE (c), and PI/GCE (d) at 5 mV/s.

The primary purpose of the present study is simultaneous detection of AA, DA, and UA. Usually, the voltammetric peaks for the oxidation of AA, DA, and UA are overlapped and hard to distinguish. To check the usefulness of the doping of CB and CNT within the PI, we compared the cyclic voltammetry (CV) of ternary mixture of AA, DA, and UA by using various electrodes. Figure 2A shows the CV responses of CB-CNT/PI/GCE (a), CB/PI/GCE (b), CNT/PI/GCE (c), and PI-GCE (d). As compared to other three electrodes, the CB-CNT co-doped PI film-modified GCE (CB-CNT/PI/GCE) (a) exhibited well-defined oxidation peaks at −100, 200, and 360 mV, corresponding to the oxidation of AA, DA, and UA, respectively. The difference in CV responses between the CB-CNT/PI/GCE (a) and the CB/PI/GCE (b) seems to correspond to the differences in dopant amount on the electrode surface and surface morphology as shown in Figure 1.

In general, differential pulse voltammetry (DPV) has been widely utilized for the simultaneous detection of AA, DA, and UA, due to high sensitivity and selectivity [28,29,30]. Figure 2B illustrates DPV responses of ternary mixture of AA, DA, and UA obtained by CB-CNT/PI/GCE (a), CB/PI/GCE (b), CNT/PI/GCE (c), and PI-GCE (d). The PI/GCE (d) did not show clear response, indicating that the doping of CB and CNT within the PI film is essential to obtain the electrochemical responses. As expected, the CB-CNT/PI/GCE (a) exhibited three well-defined anodic peaks at −40, 140, and 280 mV. These results strongly suggest the potential ability of the CB-CNT/PI/GCE for simultaneous determination of AA, DA, and UA, based on the selective electrocatalytic behavior.

### 3.5. Differential Pulse Voltammetry (DPV) for Simultaneous Detection of AA, DA, and UA

**Figure 3:**
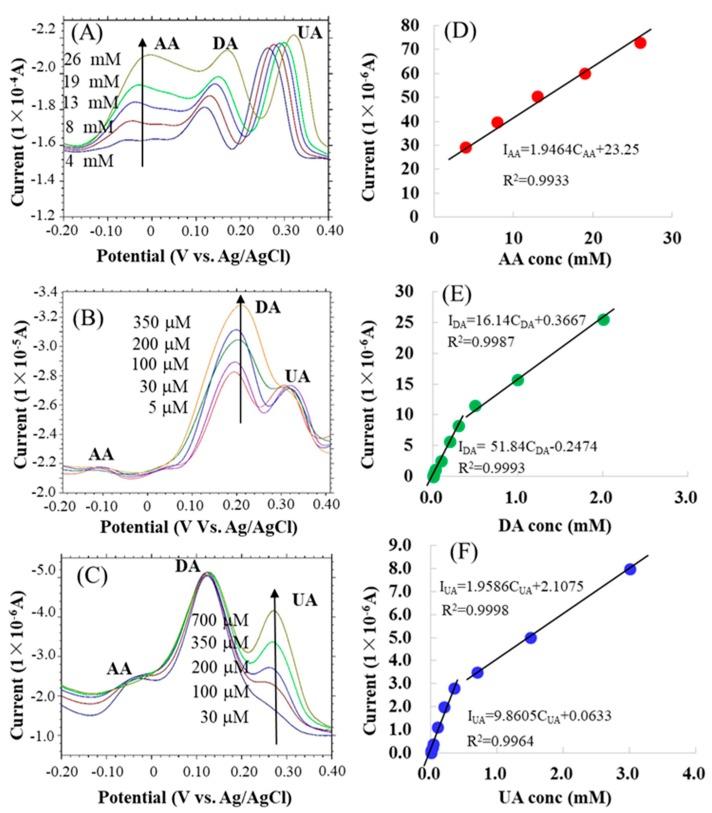
DPV curves of CB-CNT/PI/GCE in 0.1 M PBS (pH 7.0) with the AA/DA/UA mixtures: (**A**) 4−26 mM AA + 0.005 mM DA + 0.1 mM UA; (**B**) 5.0 mM AA + 5–350 μM DA + 1 mM UA; (**C**) 1.0 mM AA + 0.1 mM DA + 30–700 μM UA. (**D**–**F**) The oxidation current of substrates (I) vs. concentrations (C).

Next, we tried individual determination of each substance in the ternary mixture with DPV. Figure 3 illustrates the DPV curves (Figure 3A–C) and calibration plots (the oxidation current versus the concentration) (Figure 3D–F) obtained by the measurement of ternary mixture of AA, DA, and UA. As shown in Figure 3A–C, the oxidation peak current of AA, DA, or UA increases with the increasing of individual concentrations in the presence of the other two substrates, and the linear calculation plots are shown in Figure 3D–F. In the case of AA (Figure 3A), the broad AA peak at ca. −50 mV increased linearly with the increase in AA concentration (Figure 3D). In the case of DA (Figure 3B), the peak current at +200 mV clearly increases with the increase in DA concentration, and two linear portions can be seen in the calibration plot (Figure 3E). In a low concentration range of DA (C_DA_: 3–300 µM), the linear regression equation is I_DA_ = 51.84C_DA_ − 0.2474, with a correlation coefficient of R^2^ = 0.9993. In a high concentration range of DA (C_DA_: 300–2000 µM), the linear regression yields a relationship of I_DA_ = 16.14C_DA_ + 0.3667, with a correlation coefficient of R^2^ = 0.9987. Similar results were observed for UA (Figure 3C,F), and two linear regions are also observed in the calibration plot (Figure 3F). The linear regression equations are I_UA_ = 9.8605C_UA_ + 0.0633 (C_UA_: 1–500 μM, R^2^ = 0.9964) and I_UA_= 1.9586C_UA_ + 2.1075 (C_UA_: 500–3000 μM, R^2^ = 0.9998). The observed two slopes could be explained by monolayer adsorption followed by multilayer adsorption [31], or existence of different structural layer portion within the composite CB-CNT/PI film. The lower sensitivity in the high concentration region could be caused by limited active sites of CB and CNT for the oxidation of DA and UA [32]. As shown in Figure 3A,D, AA has only one linear region from 4 to 26 mM, probably because the second region is located in higher concentrations. The sensitivity of AA is much lower than DA and UA. This tendency has also been reported by other carbon-nanomaterials-based electrodes [31,33,34,35]. The approach of negatively-charged AA to the electrode surface through the PI film probably inhibited by the electric repulsion with negative charge of AA and carboxyl group of the graphite edge of CB and CNT.

### 3.6. Electrochemical Determination of AA, DA, and UA

**Figure 4:**
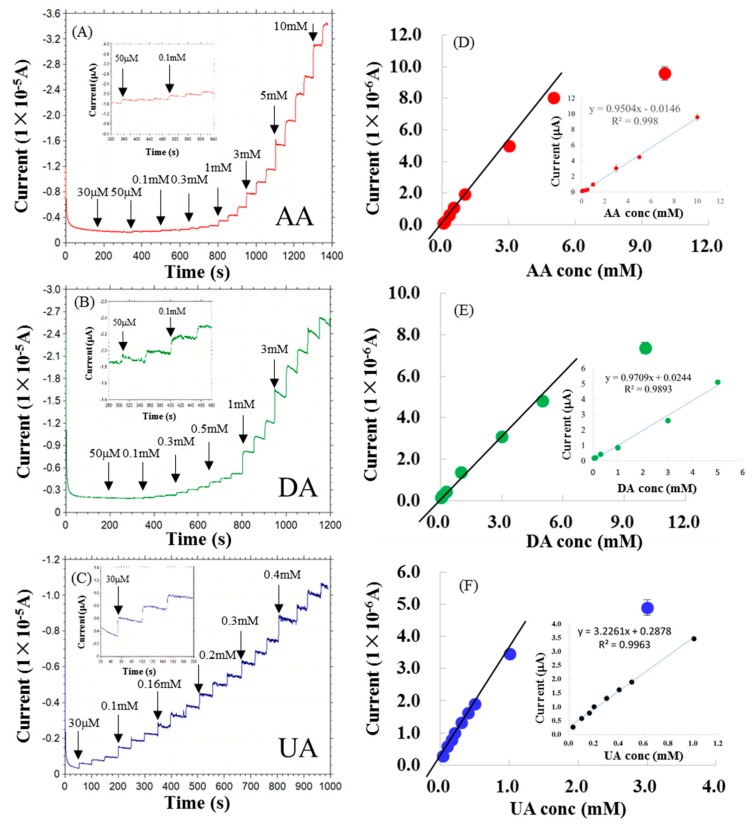
Steady-state amperometric responses of the CB-CNT/PI/GCE for the subsequent addition of (**A**) AA (−0.05 V), (**B**) DA (+0.2 V) (**C**) UA (+0.35 V) in a 0.1 M PBS (pH 7.0). The oxidation curves vs. the concentration of various substances: (**D**) AA, (**E**) DA, and (**F**) UA.

The steady-state amperometric responses of the CB-CNT/PI/GCE to AA, DA, and UA are presented in Figure 4. Different concentrations of analytes were added into a stirring 0.1 M PBS (pH 7.0) with a fixed potential. The corresponding plots of the currents and analyte concentrations are calculated in Figure 4D–F. From the amperometric curve for sensing AA in Figure 4A, the linear relationship between the oxidation current and concentration is obtained in the range of 0.1–5 mM. The linear regression equation is I_AA_= 0.9504C_AA_ − 0.0146, with a correlation coefficient of R^2^ = 0.998 (n = 3). The detection limit of AA was found to be 75 μM. Figure 4B shows the amperometric response of DA at the potential of +0.2 V. The linear range of DA is from 0.08 to 3 mM, with the linear regression equation is I_DA_ = 0.9709C_DA_ + 0.0244, a correlation coefficient of R^2^ = 0.9893 (n = 3). The detection limit of DA was found to be 60 μM. Similarly, Figure 4C exhibits the amperometric response of UA at a potential of +0.35 V. The linear relationship of UA was determined for UA concentrations ranging from 0.01 to 1 mM. The regression equation is I_UA_ = 3.2261C_UA_ + 0.2878, a correlation coefficient of R^2^ = 0.9963 (n = 3). The detection limit of UA was found to be 8.8 μM. The insets in Figure 4D–F are the calibration curves for low concentrations of AA, DA, and UA.

### 3.7. Selectivity, Reproducibility, and Long-Term Stability

Selectivity is a key factor to evaluate the performance of sensors. Effect of several potential interferential species on the determination of AA, DA, and UA were carried out. When 10-times concentration (3 mM) of FeCl_3_, NH_4_Cl, NaCl, KCl, glucose, fructose, maltose, citric acid, urea, H_2_O_2_, and Na_2_SO_4_ were added into the mixture of 0.3 mM AA, 0.3 mM DA, and 0.3 mM UA, no obvious interference can be observed (Table 1), indicating the good anti-interference ability of the CB-CNT/PI/GCE. To check the reproducibility of the electrode preparation, three electrodes were prepared under the same conditions. It was found that the relative standard deviation (RSD, n = 3) was 3.1% for these electrodes, which shows high repeatability of the fabrication method. In addition, to evaluate the practical usage of the present sensor, the long-term stability of the CB-CNT/PI/GCE was investigated by continuous detecting of these three substances every five days for a month. When not in use, the sensor was put in a laboratory in air conditioning. Even after 1 month storage, it remained 95.6% of initial current. These sufficient performances are satisfied because of the stable characteristics of PI.

### 3.8. Real Sample Analysis

The practical application for determination of AA, UA, and DA in human urine samples was carried out to check the performance of the present sensor. A fresh urine sample was prepared by 50-fold dilution of urine with PBS (pH 7). Amperometry technique was used in an electrochemical cell containing 400 μL of urine sample and 20 mL of PBS. Different concentration of AA, DA, and UA was added into the sample. Table 2 summarizes the collected data in which recovery ranged with 90.7% to 108.2%. The results indicate that the CB-CNT/PI/GCE can be applied successfully for the electrochemical detection of AA, DA, and UA in real biological samples. The PI would effectively suppress the biofouling of the electrode surface, and allows the detection in body samples, which is one of the notable advantages of this system. Table 3 summarizes the analytical performance of various electrodes for the electrochemical determination of AA, DA, and UA.

## 4. Conclusions

The CB and CNT-co-doped PI was modified on the glassy carbon electrode, and as-prepared electrode (CB-CNT/PI/GCE) was successfully utilized for the simultaneous detection of AA, UA, and DA. The CB-CNT/PI/GCE exhibited high electrocatalytic activities towards the oxidation of AA, DA, and UA, and sufficient peak separation between AA, DA, and UA was observed with CV and DPV. The CB-CNT/PI/GCE showed sufficient selectivity and stability, and was applicable to the analysis in a human urine sample. The large capacity of PI and high surface area of CB and CNT make it a promising candidate for electrocatalytic applications and biomedical applications.

## Figures and Tables

**Table 1:** Results of interference study on the response of ascorbic acid (AA), dopamine (DA), and uric acid (UA).

Interferent	Current Intensity Variation (AA)	Current Intensity Variation (DA)	Current Intensity Variation (UA)
FeCl_3_	0.99%	2.61%	0.69%
NH_4_Cl	−2.63%	−6.29%	−1.82%
NaCl	0.74%	2.39%	0.51%
KCl	0.63%	1.66%	0.43%
Urea	0.43%	1.26%	0.65%
Citric acid	0	0	0
Glucose	0	0	0
Fructose	0	0	0
Maltose	0	0	0
H_2_O_2_	0	0	0
Na_2_SO_4_	0	0	0

**Table 2:** Determination of AA, DA, and UA in human urine samples (n = 3).

Sample		Add (μmol L^−1^)	Found (μmol L^−1^)	RSD (%)	Recovery (%)
Urine 1	AA	200	204.0	2.5	102.0
	DA	100	107.5	1.9	108.0
	UA	50	49.9	3.2	99.8
Urine 2	AA	200	195.8	2.4	97.9
	DA	100	90.7	3.2	90.7
	UA	50	52.1	3.5	104.2
Urine 3	AA	200	192.8	2.8	96.4
	DA	100	107.4	2.7	107.4
	UA	50	54.1	3.1	108.2

**Table 3:** Comparison of analytical performances the carbon black-carbon nanotube (CB-CNT)/polyimide (PI)/glassy carbon electrode (GCE) with other reported materials for electrochemical determination of AA, DA, and UA.

Electrode	Method	Linear Range (µM)			Detection Limit (µM)			Peak Separation (mV)		Refs.
		AA	DA	UA	AA	DA	UA	AA-DA	DA-UA	
**ERGO/GCE ^a^**	**DPV**	300–2000	0.5–60	0.5–60	300	0.5	0.5	240	130	[33]
MoS_2_/PEDOT/GCE ^b^	DPV	20–140	1–80	2–25	5.83	0.52	0.95	210	150	[9]
MWCNT/PEDOT ^c^	DPV	100–2000	10–330	10–25	100	10	10	220	140	[34]
ZnO/RM/GCE ^d^	CV	15–240	6–960	50–800	1.4	0.7	4.5	----	----	[36]
Fe_3_O_4_/rGO/GCE ^e^	DPV	1000–9000	0.5–10	----	0.12	0.42	----	210	----	[37]
nanoSnO_2_/MWCNTs/CPE ^f^	DPV	0.1–5	0.3–50	3–200	50	0.03	1.0	224	165	[38]
Pt-Gr-CNT/GCE ^g^	DPV	200–900	0.2–30	0.1–50	50	0.01	0.1	208	140	[35]
GE/Au/GE/CFE ^h^	DPV	----	0.6–44.0	12.6–413.6	----	0.6	12.6	108	150	[39]
CB-CNT/PI/GCE	DPV	1000–24000	3–300	5–500	154	1.86	3.0	166	148	This work
	amperometry	100–5000	80–3000	10–1000	75	60	8.8	----	-----	

^a^ electrochemically reduced graphene oxide/Glassy carbon electrode (GCE); ^b^ MoS_2_/PEDOT nanocomposite modified GCE; ^c^ MWCNT-PEDOT film modified electrode; ^d^ zinc oxide/redox mediator modified GCE; ^e^ chitosan-graphene modified GCE; ^f^ SnO_2_ nanoparticles/multi-walled carbon nanotubes/carbon paste electrode; ^g^ platinum nanoparticle-decorated graphene and carbon nanotube; ^h^ graphene sheets and gold nanoparticles modified carbon fiber electrode; differential pulse voltammetry (DPV); cyclic voltammetry (CV).

## Supplementary Materials

The following are available online at http://www.mdpi.com/1996-1944/11/9/1691/s1, Figure S1: Synthesis of dinitro compound and diamine monomer, Figure S2: ^1^H-NMR spectra of 9,9′-Bis[4-(4-nitro-2-hydroxybenzoxyl) phenyl] fluorene (BNHOPF), Figure S3: ^1^H-NMR spectra of 9,9′-Bis[4-(4-amino-2-hydroxybenzoxyl) phenyl] fluorene (BAHOPF), Figure S4: Synthesis procedures of polyimide; Figure S5: FT-IR spectras of dinitro compound (**a**) and diamine monomer(**b**), Figure S6: FT-IR of polyimide, Figure S7: XRD of polyimide.
