# Supplementary material for: Carbon Black-Carbon Nanotube Co-Doped Polyimide Sensors for Simultaneous Determination of Ascorbic Acid, Uric Acid, and Dopamine
Source: Materials (Basel). 2018 Sep 12;11(9):1691. doi: 10.3390/ma11091691 (PMC6163399; doi:10.3390/ma11091691)
Supplement: Supplementary file 1 [file materials-11-01691-s001.pdf]

materials

Supplementary: Carbon Black-Carbon Nanotube Co-Doped Polyimide Sensors for Simultaneous Detection of Ascorbic Acid, Uric Acid, and Dopamine

Yue Wang, Tian Yang, Yasushi Hasebe, Zhiqiang Zhang and Dongping Tao

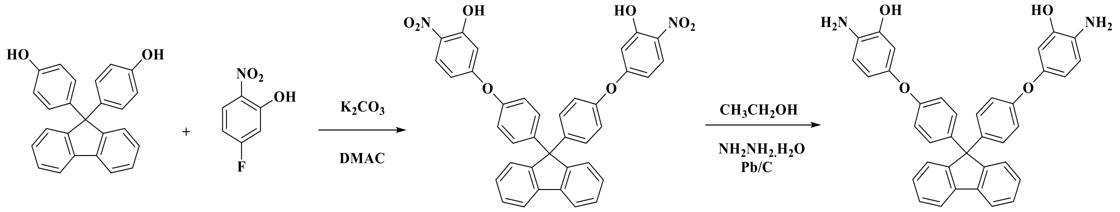

Figure S1. Synthesis of dinitro compound and diamine monomer.

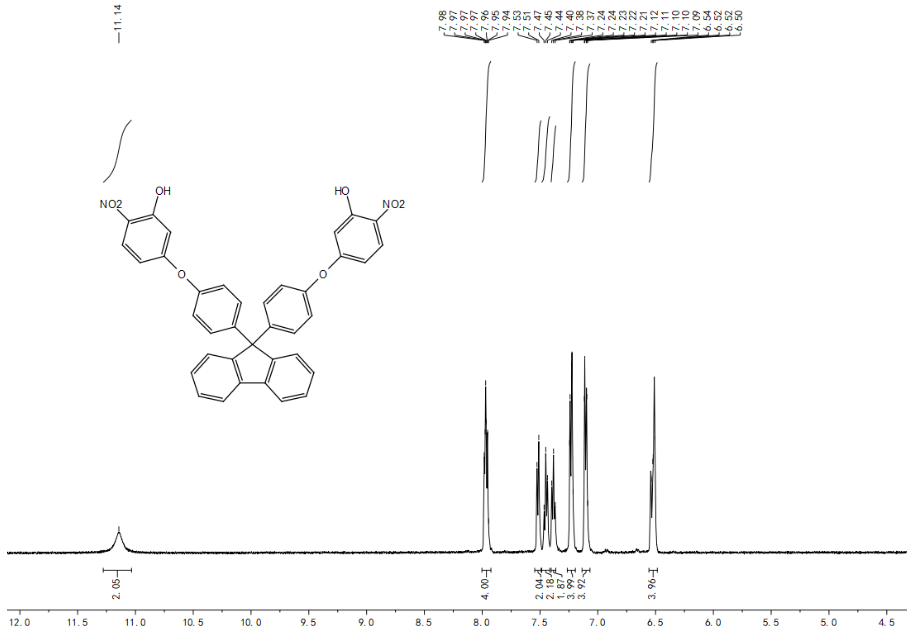

Figure S2. ^{1}H-NMR spectra of 9, 9′-Bis[4-(4-nitro-2-hydroxybenzoxyl) phenyl] fluorene (BNHOPF).

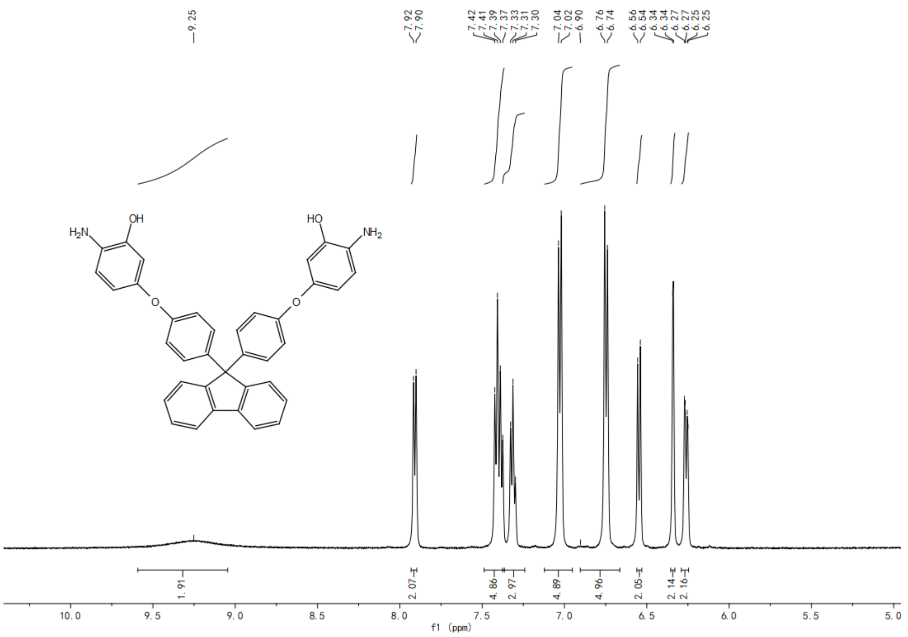

Figure S3. ^{1}H-NMR spectra of 9,9′-Bis[4-(4-amino-2-hydroxybenzoxyl) phenyl] fluorene (BAHOPF).

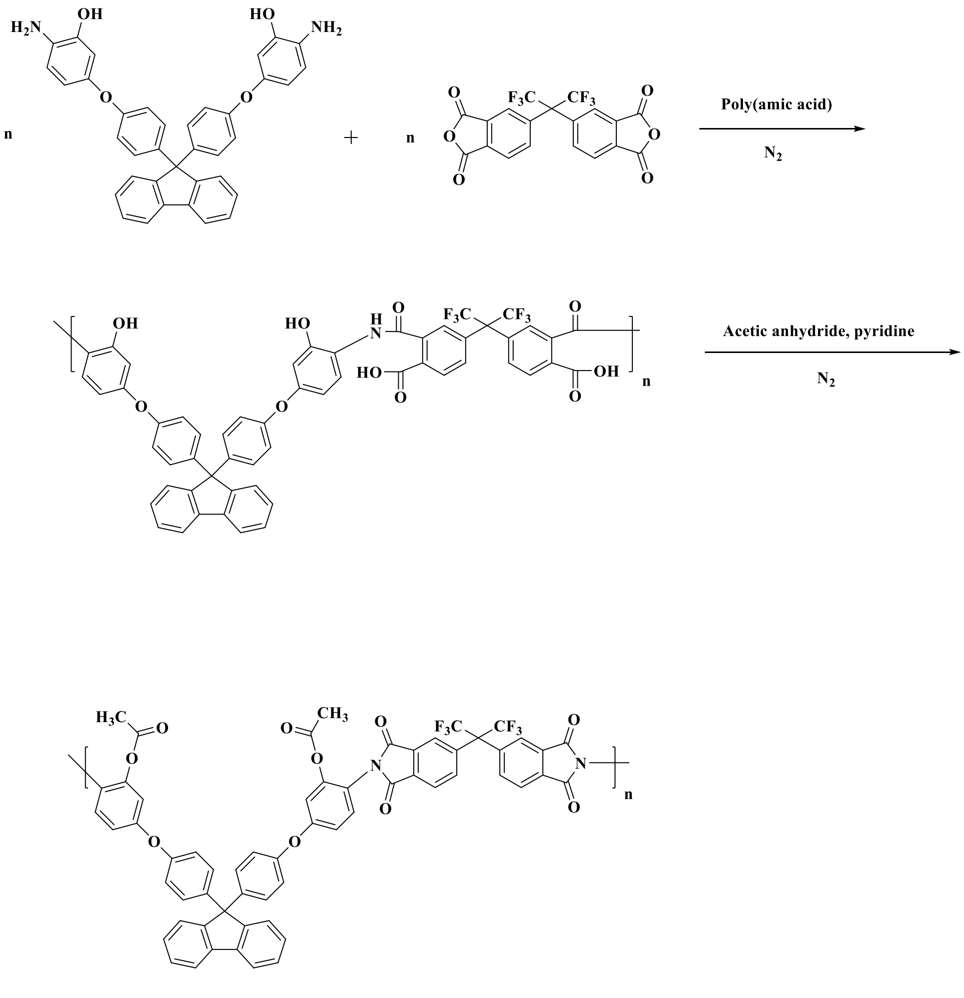

Figure S4. Synthesis procedures of polyimide.

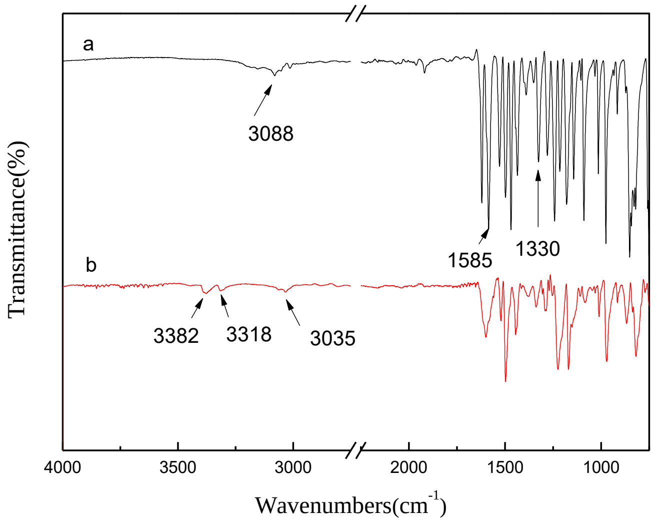

Figure S5. FT-IR spectras of dinitro compound (a) and diamine monomer(b).

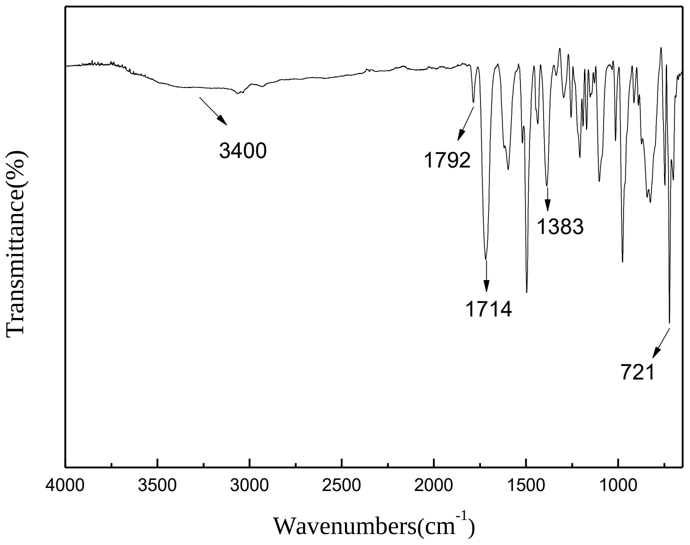

Figure S6. FT-IR spectra of polyimide.

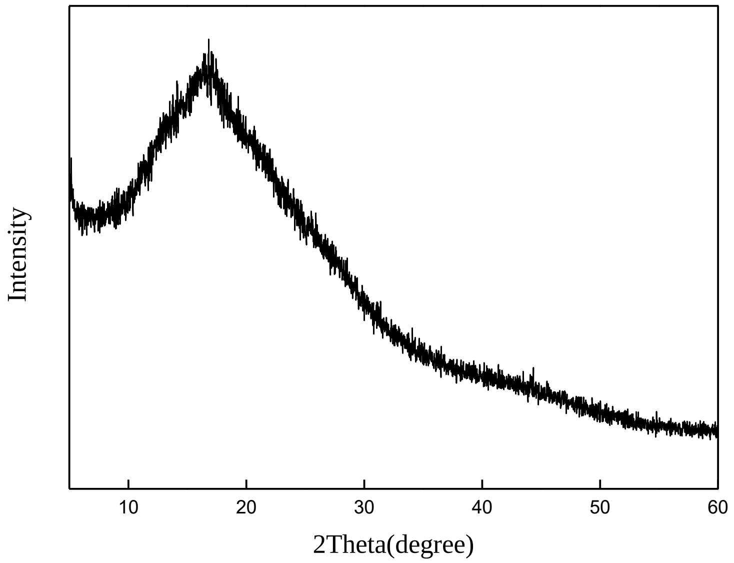

Figure S7. XRD patterns of polyimide.

© 2018 by the authors. Submitted for possible open access publication under the terms and conditions of the Creative Commons Attribution (CC BY) license (http://creativecommons.org/licenses/by/4.0/).
